# Real‐time monitoring of seroma changes during breast cancer radiotherapy using Cherenkov imaging: A technical note

**DOI:** 10.1002/acm2.70553

**Published:** 2026-05-10

**Authors:** Michael Tallhamer, Adi Robinson

**Affiliations:** ^1^ Department of Radiation Oncology AdventHealth Parker Parker Colorado USA; ^2^ Department of Radiation Oncology AdventHealth Celebration Celebration Florida USA

**Keywords:** adaptive radiotherapy, breast cancer, Cherenkov imaging, seroma, SGRT

## Abstract

**Background:**

Seroma formation after breast‐conserving surgery is a common postoperative occurrence that can significantly affect the accuracy of radiation therapy. Variations in seroma size and shape during treatment introduce uncertainties in dose delivery, potentially compromising target coverage and increasing exposure to healthy tissue. Conventional monitoring relies on periodic imaging such as CT or MRI, which may not capture dynamic changes during the treatment course.

**Purpose:**

This technical note investigates the feasibility of using Cherenkov imaging for real‐time monitoring of seroma evolution during breast cancer radiotherapy. The goal is to determine whether this approach can provide actionable information to improve treatment accuracy and support adaptive planning.

**Methods:**

Breast cancer patients undergoing whole‐breast irradiation were treated using a TrueBeam linear accelerator (Varian, Palo Alto, CA) with surface guided radiation therapy (SGRT) for positioning and breath‐hold gating. Seromas were initially contoured during treatment planning and reassessed mid‐treatment using cone‐beam CT. Cherenkov imaging (DoseRT, VisionRT) was integrated into daily treatment sessions to visualize seroma changes in real time. Seroma size and shape were recorded for each fraction and compared with initial planning volumes and mid‐treatment imaging.

**Results:**

Cherenkov imaging successfully identified and tracked seroma changes throughout the treatment course. Significant variability was observed among patients: some exhibited progressive enlargement, while others demonstrated resolution. For example, one patient's seroma increased from 24 cm^3^ at planning to 29 cm^3^ at completion, whereas another decreased from 12.5 to 5.8 cm^3^. Pixel‐based measurements from Cherenkov imaging correlated with volumetric changes observed on CT, confirming the reliability of this technique. These findings underscore the dynamic nature of seromas and the potential need for adaptive interventions during treatment.

**Conclusions:**

Cherenkov imaging offers a practical, noninvasive solution for real‐time monitoring of seroma changes during breast cancer radiotherapy. By providing immediate feedback on anatomical variations, this technology can enhance treatment accuracy, reduce unnecessary radiation exposure to healthy tissues, and inform adaptive planning strategies. Further research is warranted to evaluate clinical outcomes, workflow integration, and cost‐effectiveness.

## 1 INTRODUCTION

Breast cancer is among the most prevalent malignancies in women, and radiation therapy is essential for breast conserving treatment. Radiation therapy is critical in reducing the risk of local recurrence following breast‐conserving surgery.^1^ However, one of the challenges in delivering effective radiation therapy is the presence of postsurgical complications such as seroma formation.

A seroma is an accumulation of fluid in the cavity left after the surgical removal of a tumor. While seromas are a common postoperative occurrence, their presence can significantly impact the accuracy and effectiveness of radiation therapy.^2^, ^3^ The dynamic nature of seromas, which can change in size and shape over time, poses a challenge to maintaining precise targeting during the treatment course. Variations in the size and location of the seroma can lead to discrepancies between the planned radiation dose and the actual dose delivered to the tumor bed, potentially affecting treatment outcomes.^4^, ^5^

Traditional methods of monitoring seromas during radiation therapy involve periodic imaging with techniques such as computed tomography (CT) or magnetic resonance imaging (MRI). Adaptive radiotherapy (ART) could potentially offer real‐time data and replanning assistance but that is yet to be explored.^6^ The delay between imaging sessions can result in inaccuracies, as changes in the seroma's size and position may not be promptly accounted for in the radiation therapy plan.

Cherenkov imaging has recently emerged as an innovative technique in radiation oncology, offering real‐time visualization of radiation dose distribution and anatomical changes during treatment.^7^, ^8^ Cherenkov radiation occurs when charged particles move faster than the speed of light in a dielectric medium, emitting light that can be captured and visualized.^9^ This imaging technique enables clinicians to monitor the dynamic changes in the treatment area, including the seroma, during each radiation session.

Prior work has demonstrated the feasibility of Cherenkov imaging for real‐time visualization of radiation delivery and surface dose patterns, including inter‐fraction and intra‐fraction consistency during external beam radiation therapy.^7^, ^8^, ^10^, ^11^ However, the application of Cherenkov imaging for longitudinal monitoring of evolving anatomical features during a treatment course, such as postsurgical seromas in breast cancer patients, has not been characterized. In particular, the potential role of Cherenkov imaging as a real‐time screening and decision‐support tool to identify clinically meaningful anatomical changes that may warrant further imaging or adaptive intervention remains underexplored.

To date, clinical applications of Cherenkov imaging have primarily focused on visualization of delivered dose and surface dose patterns. The use of Cherenkov imaging for segmentation and monitoring of evolving anatomical features during a treatment course has not previously been reported.

This study explores the use of Cherenkov imaging to monitor seroma changes in breast cancer patients undergoing radiation therapy. By providing real‐time data, Cherenkov imaging has the potential to improve the accuracy of radiation delivery, minimize exposure to healthy tissues, and enhance clinical decision‐making throughout the course of treatment.

## 2 MATERIALS AND METHODS

A total of 6 breast cancer patients were selected for this study, comprising 3 patients with left‐sided breast cancer and 3 patients with right‐sided breast cancer. Each patient underwent radiation therapy using a TrueBeam linear accelerator (Varian, Palo Alto, CA). Patient setup and positioning were facilitated by AlignRT (VisionRT, London, UK), a Surface guided radiation therapy (SGRT) system that provides real‐time feedback on patient positioning to ensure sub‐millimeter accuracy. The use of SGRT was particularly beneficial in cases where patients were treated with the breath‐hold technique, as AlignRT was employed to gate the radiation beam based on the patient's respiratory cycle, further enhancing the precision of dose delivery. Patient positioning was verified with Image guidance radiation therapy (IGRT) using the on‐board imager on the Truebeam linear accelerator.

The ability to continuously monitor patient positioning throughout each fraction was integral to maintaining the consistency and reproducibility of the radiation delivery. This high level of precision ensured that the radiation fields were accurately aligned with the planned target volumes, mitigating the risk of geographic miss or unnecessary exposure to adjacent healthy tissues.

The seroma was initially contoured as part of the lumpectomy at the time of treatment planning for every patient in this study. A cone‐beam CT (CBCT) was used at fraction 15 to check the size of the seroma and monitor changes. Seroma changes were also monitored in real‐time with DoseRT (VisionRT, London, UK), a Cherenkov imaging system designed to capture and display the Cherenkov light emitted during radiation delivery. The use of Cherenkov imaging allowed us to dynamically visualize the seroma during each treatment fraction, tracking its size and shape to detect any significant changes that might necessitate adjustments to the treatment plan. Using Cherenkov imaging each patient's seroma was identified, monitored and evaluated after each fraction. As part of the evaluation, the prescribing physician was consulted, and any modifications to the treatment plan followed standard clinical procedures.

Seroma regions were identified on Cherenkov images based on localized, reproducible regions of altered Cherenkov signal intensity corresponding spatially to the lumpectomy cavity projected onto the patient surface. Initial identification was performed using the cumulative Cherenkov image from the first delivered fraction, which provides a high signal‐to‐noise representation of the delivered radiation field.

Seroma segmentation was performed manually by a medical physicist with experience in Cherenkov imaging and breast radiotherapy, with confirmation of baseline identification by the treating physician. Contours were drawn using a freehand region‐of‐interest tool within the DoseRT analysis environment. The same observer performed all segmentations for a given patient to ensure internal consistency across fractions.

No automated thresholding, edge‐detection, or machine learning–based segmentation was employed in this study. Inter‐ and intra‐observer variability were not formally assessed, as the primary goal of this technical note was to evaluate feasibility and workflow integration rather than contouring precision.

Quantitative measurements of seroma size were derived from cumulative Cherenkov images generated for each treatment fraction by integrating all valid frames acquired during beam‐on time. For patients treated using deep inspiration breath hold (DIBH), only frames meeting the SGRT gating criteria were included in the cumulative image, minimizing the influence of respiratory motion.

The reported seroma size represents the two‐dimensional projected area of the manually contoured region on the Cherenkov image plane. These measurements were used to assess relative changes in seroma appearance over the course of treatment rather than absolute volumetric dimensions.

Cherenkov images acquired during radiation delivery represent two‐dimensional optical projections of emitted light from the patient's surface. As such, the apparent size of anatomical features in the image is influenced by camera position, patient‐to‐camera distance, viewing angle, and surface curvature. Although the DoseRT system uses a fixed camera geometry for each treatment unit, direct conversion of pixel‐based area measurements to physical units (e.g., mm^2^) is non‐trivial and patient dependent.

For this reason, seroma area measurements in this study are reported in pixel‐squared units and interpreted as relative, intra‐patient metrics rather than absolute physical dimensions. Longitudinal comparisons were performed within individual patients under consistent imaging geometry, allowing meaningful assessment of temporal trends.

This combination of SGRT and Cherenkov imaging provided a comprehensive approach to patient management, allowing for real‐time adjustments that enhance the precision and efficacy of radiation therapy.

Cherenkov images were acquired using the DoseRT optical imaging system with fixed camera geometry. Image acquisition parameters, including camera integration time and frame averaging, were consistent across all patients. Ambient room lighting was controlled during image acquisition to minimize background signal, and standard system normalization procedures were applied prior to analysis.

## 3 RESULTS

**FIGURE 1:**
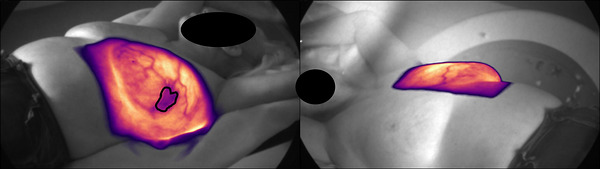
Cumulative Cherenkov image acquired after completion of a treatment fraction, displayed in the camera viewing orientation. The manually contoured seroma region is outlined in black. The image represents the integrated Cherenkov signal from all gated frames delivered during beam‐on time.

**FIGURE 2:**
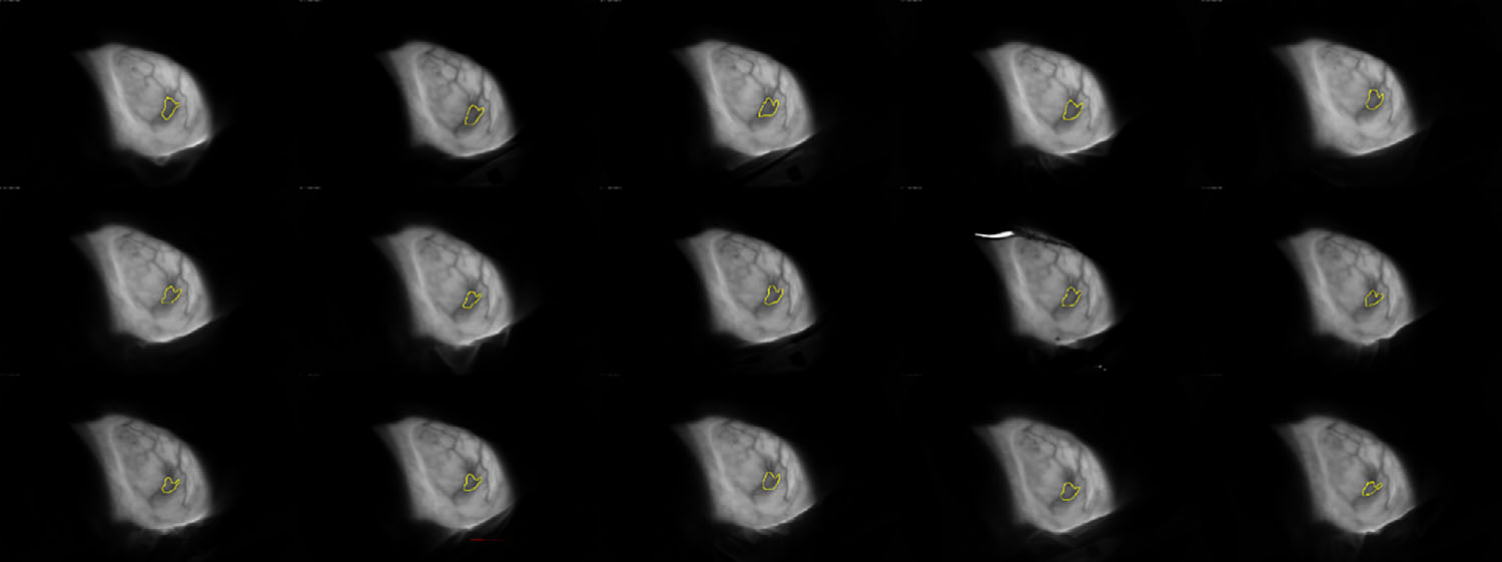
Longitudinal seroma monitoring over a 15‐fraction left breast radiation treatment course using cumulative Cherenkov imaging. The seroma is manually contoured in yellow on the cumulative image for each fraction, illustrating changes in projected area over time.

At the start of each radiation course, we evaluated the cumulative Cherenkov image of the first fraction delivered. That image, as seen in Figure 1, was used to verify the delivered fraction and identify the presence of a seroma. Once a seroma is identified, it is continuously monitored for subsequent fractions until the radiation course is fully delivered. Figure 2 shows the changes a seroma might present during a standard left breast 15 fraction course of radiation. The seroma can also be contoured and carefully monitored for changes in shape and size, as seen in Figure 2. Table 1 shows the volume of the seroma at the start and end of the radiation treatment. Table 2 shows the measured area of a seroma over the course of a 15‐fraction radiation treatment using Cherenkov imaging for several patients. As an example, patient 1 had a seroma size increase at fraction 11 that required adjustment to the treatment plan.

**TABLE 1:** Seroma volume change from CT planning to last delivered fraction for each patient in this study. Seroma size is given in centimeters cubed.

Fraction	Patient 1	Patient 2	Patient 3	Patient 4	Patient 5	Patient 6
0	24	8.6	18	16.6	12.5	9.3
10	–	–	–	13.3	–	–
15	29	9.2	21.2	–	5.8	8.4

**TABLE 2:** Seroma size changes per fraction for each patient in this study. Seroma size is given in pixels squared.

Fraction	Patient 1	Patient 2	Patient 3	Patient 4	Patient 5	Patient 6
1	1574	960	2259	1209	666	941
2	1676	1143	2247	1180	713	877
3	1690	1064	2198	1258	714	848
4	1596	1034	2610	1138	699	881
5	1681	1055	2677	1098	509	956
6	1693	1072	2689	1086	463	876
7	1633	1081	2549	1075	425	912
8	1694	1121	2802	1026	433	868
9	1537	1092	2878	899	452	914
10	1509	1071	2890	871	495	820
11	1856	1033	2994		481	805
12	2078	1042	2720		382	871
13	2295	1065	2769		437	816
14	2330	1099	2889		490	904
15	2480	1082	2890		385	859

Across the cohort, longitudinal trends in Cherenkov‐derived seroma area were compared qualitatively with volumetric changes observed on CT imaging acquired at planning and during treatment. In five of six patients, the directionality of change (increase or decrease) in projected Cherenkov area was concordant with the corresponding change in CT‐derived seroma volume. Although the limited sample size precluded formal statistical testing, this agreement supports the hypothesis that Cherenkov‐based area trends reflect underlying anatomical evolution of the seroma during treatment.

## 4 DISCUSSION

The addition of Cherenkov imaging into daily radiation therapy treatment represents a substantial advancement in the management of patients with breast cancer, particularly for those who develop postsurgical seromas. Traditionally, radiation therapy plans are based on pretreatment imaging, with assumptions that the internal anatomy will remain relatively stable throughout the course of treatment. However, the presence and variability of seromas introduce significant uncertainties into this process, as seromas can change in both size and shape over time, leading to potential discrepancies between the planned and delivered radiation doses.

In this study, Cherenkov imaging was used as a screening and decision‐support tool rather than as an autonomous trigger for adaptive replanning. A seroma change was considered clinically significant when sustained changes in projected area exceeding approximately 20% were observed across multiple fractions, when visible deformation or migration relative to the planned lumpectomy cavity was noted, or when qualitative concern was raised during physician review. When these criteria were met, actions included physician evaluation, repeat CBCT acquisition, and reassessment of target contours. In one case (Patient 1), confirmation of seroma expansion at fraction 11 led to a mid‐course treatment plan adjustment. These criteria were applied retrospectively and are presented as a proposed framework for future protocol‐driven workflows.

Because Cherenkov imaging provides a two‐dimensional surface‐projected representation of emitted light, apparent changes in seroma area may be influenced by patient setup variability or subtle changes in posture. The use of SGRT‐based positioning and gated acquisition mitigates, but does not eliminate these effects. Importantly, the observed seroma trends were typically consistent across multiple consecutive fractions, reducing the likelihood that setup variation alone accounts for the observed changes.

Cherenkov imaging offers a novel solution to these challenges by providing real‐time visualization of the treatment area during each radiation treatment. This enables clinicians to directly observe the size and shape of the seroma during each treatment session, offering insight into the daily anatomical variations that occur in the patient's body.

Furthermore, the ability to observe the seroma in real‐time can provide critical information for decision‐making during treatment. For example, if the seroma is observed to be shrinking significantly during the course of therapy, it may be possible to adjust the treatment plan to more accurately conform to the reduced target volume. Conversely, if the seroma remains stable or increases in size, clinicians can use this information to make informed decisions about the need for additional imaging, potential treatment breaks, or modifications to the treatment plan.

The data presented in Tables 1 and 2 demonstrate the variability in seroma size across multiple treatment fractions for each patient. Overall, patients exhibited distinct patterns of seroma volume change, underscoring the dynamic nature of seroma evolution during radiation therapy. For instance, Patient 1 experienced an increase in seroma size from fraction 1 (1574 pixels squared) to fraction 15 (2480 pixels squared), indicating gradual accumulation over time. This increase in size showed correlation with the increase in volume from fraction 0 (24 cc) to fraction 15 (29 cc). Conversely, Patient 5 showed a marked decrease from 666 pixels squared in fraction 1 (12.5 cc in fraction 0) to 385 pixels squared by fraction 15 (5.8 cc in fraction 15), suggesting resolution of the seroma over the course of treatment. Patients 3 and 6 also displayed fluctuations, with initial increases in seroma size that eventually stabilized or decreased. These variations highlight the importance of real‐time monitoring with Cherenkov imaging, as seroma changes could significantly impact the accuracy of radiation dose delivery. By identifying seroma volume trends throughout the treatment course, clinicians may be better equipped to adapt the treatment plan, potentially leading to improved targeting accuracy and minimizing radiation exposure to surrounding healthy tissue.

Traditional radiation therapy relies on static imaging acquired at the beginning of treatment, which may not accurately reflect the patient's anatomy throughout the course of therapy. This can lead to unintentional radiation exposure to healthy tissues, particularly if the seroma shifts or changes in size during treatment. By providing real‐time visualization of the treatment area, Cherenkov imaging allows for more precise targeting, potentially reducing the dose to surrounding healthy tissues and lowering the risk of side effects such as radiation pneumonitis, fibrosis, or cardiac toxicity.

In addition to its clinical benefits, Cherenkov imaging also offers practical advantages in a radiation oncology workflow. The technology is noninvasive and can be seamlessly integrated into existing treatment protocols without requiring additional imaging sessions or significant changes to the patient's treatment schedule. This ease of integration ensures that the benefits of Cherenkov imaging can be realized without adding to the patient's burden or the department's workload.

Future development efforts will focus on improving the efficiency and scalability of Cherenkov‐based seroma monitoring, including semi‐automated or machine learning–assisted segmentation, surface‐mapped geometric correction using SGRT data, and integration into ART decision frameworks. These advances could further enhance the clinical utility of Cherenkov imaging as a real‐time monitoring tool during breast radiotherapy.

## 5 CONCLUSION

In summary, the use of Cherenkov imaging in breast cancer radiation therapy represents a significant step forward in the quest for more precise, adaptable, and patient‐centered treatment. By enabling real‐time monitoring of seroma changes, this technology enhances the accuracy of radiation dose delivery, reduces the risk of treatment‐related toxicity, and provides valuable data for both clinical decision‐making and research. As the field of radiation oncology continues to evolve, Cherenkov imaging is likely to play an increasingly important role in improving the outcomes and quality of life for breast cancer patients.

## AUTHOR CONTRIBUTION

All authors contributed to the study conception and design. Material preparation, data collection, and analysis were performed by both authors. The first draft of the manuscript was written by Adi Robinson, and all authors commented on previous versions of the manuscript. All authors read and approved the final manuscript.

## CONFLICT OF INTEREST STATEMENT

**Michael Tallhamer**: Consulting fees—VisionRT—Our institution is a Center of Excellence for the vendor, and it receives payments for site visits.

Support for attending meetings and/or travel—VisionRT—Invited to speak at multiple conferences.

Receipt of equipment, materials, drugs, medical writing, gifts or other services—VisionRT—Our institution is a Center of Excellence for the vendor and it receives equipment at reduced cost for evaluation purposes as part of a Professional Services Agreement.

**Adi Robinson**: Consulting fees—VisionRT—Our institution is a Center of Excellence for the vendor, and it receives payments for site visits.

Support for attending meetings and/or travel—VisionRT—Invited to speak at multiple conferences.

The authors declare that they have no conflicts of interest/competing interests.

## ETHICS STATEMENT

Due to the retrospective nature of this study, the requirement for informed consent was waived by the hospital's IRB.

## Data Availability

Research data are stored in an institutional repository and will be shared upon request to the corresponding author.
